# Underlying Cryptococcal Diseases and the Correlation With Serum Cryptococcal Antigen Titers in Hospitalized HIV-Infected Patients Screened Positive for Cryptococcal Antigenemia

**DOI:** 10.3389/fcimb.2020.00170

**Published:** 2020-04-24

**Authors:** Miaomiao Xu, Zhihang Peng, Chuanjun Xu, Yaling Chen, Jian Cheng, Yun Chi, Hongxia Wei, Wei Chen, Zhiliang Hu

**Affiliations:** ^1^Department of Infectious Disease, The Second Hospital of Nanjing, Nanjing University of Chinese Medicine, Nanjing, China; ^2^School of Public Health, Nanjing Medical University, Nanjing, China; ^3^Department of Radiology, The Second Hospital of Nanjing, Nanjing University of Chinese Medicine, Nanjing, China; ^4^Department of Clinical Research Center, The Second Hospital of Nanjing, Nanjing University of Chinese Medicine, Nanjing, China; ^5^Center for Global Health, School of Public Health, Nanjing Medical University, Nanjing, China

**Keywords:** cryptococcal antigenemia, HIV, cryptococcal meningitis, pulmonary cryptococcosis, lateral flow assay

## Abstract

**Background:** The prevalence of different underlying cryptococcal diseases in human immunodeficiency virus (HIV)-infected patients screened positive for cryptococcal antigenemia and the association between cryptococcal diseases and serum cryptococcal antigen (CrAg) titers were understudied.

**Methods:** HIV-infected patients with CD4 < 200 cells/ul, admitted to the second hospital of Nanjing, Nanjing, China, from January 2016 to September 2019, were retrospectively analyzed. Integrated into routine HIV care, all these patients were screened for cryptococcal antigenemia with CrAg lateral flow assay. Positive patients received extensive laboratory and radiological studies to evaluate underlying cryptococcal diseases.

**Results:** A total of 872 HIV inpatients were screened for serum CrAg. The prevalence of cryptococcal antigenemia in the study population was 10.3% (95% CI, 8.3–12.3%), 87.6% of which with cryptococcal antigenemia had clinically cryptococcal diseases. The prevalence of cryptococcal meningitis (CM), cryptococcemia and pulmonary cryptococcosis (PC) in patients with cryptococcal antigenemia were 58.4% (95% CI, 48.0–68.9%), 50.7% (95% CI, 39.1–62.2%), and 68.5% (95% CI, 58.7–78.4%), respectively. The median (range) serum CrAg titers in severe cryptococcal diseases (CM or cryptococcemia), localized PC (without co-existing CM or cryptococcemia) and isolated cryptococcal antigenemia were 1:2560 (1:10–1:2560), 1:20 (1:2–1:320), and 1:5 (1:2–1:320), respectively. Serum CrAg titers ≥1:320 were independently associated with CM (adjusted OR 26.88; 95%CI, 8.36–86.42). Severe cryptococcal diseases were found in all patients with serum CrAg titers ≥1:640. None of the patients with serum CrAg titers ≤ 1:5 had CM.

**Conclusion:** The prevalence of cryptococcal antigenemia was high in HIV inpatients, supporting routine CrAg screening. Clinical cryptococcal diseases, most commonly the PC, existed in the majority of the patients with cryptococcal antigenemia. Since serum CrAg titer is correlated with cryptococcal disease severity, it may possibly guide anti-fungal treatment.

## Background

Cryptococcal diseases are common opportunistic infections in human immunodeficiency virus (HIV)-infected patients. The reported deaths of cryptococcal diseases are generally related to cryptococcal meningitis (CM) which had around 0.22 million incident cases leading to about 0.18 million deaths each year (Rajasingham et al., 2017). Cryptococcal antigenemia precedes symptoms of CM by a median of 22 days (French et al., 2002), suggesting that cryptococcal antigenemia screening may identify early cryptococcal diseases before progressing to CM. This screening strategy has been advocated in routine HIV care and may reduce mortality of HIV-infected patients (Rajasingham et al., 2012; Kaplan et al., 2015; Mfinanga et al., 2015). Recently studies calculated that the prevalence of cryptococcal antigenemia among HIV-infected outpatients with a CD4 cell count <100 was around 6.0% (Rajasingham et al., 2017; Ford et al., 2018; Temfack et al., 2019). Of note, substantial variation of the prevalence existed in different regions, with the highest prevalence reported in Ethiopia (Oromia Region, 15.5%) and lowest in Malawi (1.7%) (Rajasingham et al., 2017). The prevalence of cryptococcal antigenemia was higher among inpatients 9.8%, with also region-related variations (95% CI, 4.0–15.5%) (Ford et al., 2018).

Although the prevalence of cryptococcal antigenemia in HIV-infected out-patients has been widely explored and to a lesser extend in hospitalized HIV patients, the underlying clinical cryptococcal diseases contributing to positive serum cryptococcal antigen (CrAg) and the correlation between different cryptococcal diseases and serum CrAg titers have not been thoroughly studied. The common diagnostic procedure following a positive serum CrAg screening test is lumbar puncture to rule out CM (Ganiem et al., 2014; Mfinanga et al., 2015; Pac et al., 2015; Longley et al., 2016; Mamuye et al., 2016; Vidal et al., 2016; Beyene et al., 2017; Nalintya et al., 2018; Chen et al., 2019). Of note, cryptococcal infection generally begins with inhalation of fungal cells (May et al., 2016). However, it is unclear to what extent pulmonary cryptococcosis (PC) contributes to cryptococcal antigenemia. In the present study, we aimed to determine the prevalence of cryptococcal antigenemia, and to analyze the underlying cryptococcal diseases as well as their correlations with serum CrAg titers in Chinese hospitalized HIV-infected patients.

## Methods

### Patients

Hospitalized HIV-infected patients with CD4 cell count <200 cells/uL, at the department of infectious diseases, second hospital of Nanjing, Nanjing, China, from January 2016 to September 2019, were retrospectively analyzed. As integrated in routine care, all these patients (HIV infected, CD4 <200 cells/uL) were routinely tested for serum CrAg with lateral flow assay (IMMY Diagnostics, Norman, USA) regardless the symptoms and the severity of the of clinical illness. Patients with positive serum CrAg tests received lumbar punctures, blood cultures and chest CT scans to search for possible clinically relevant cryptococcal diseases. Treatments of the cryptococcal diseases were in accordance with clinical practice guidelines (Perfect et al., 2010). The demographic, clinical and laboratory data of the patients were collected from an electronic health record system. The data were analyzed and presented anonymously. Patients with previous treated cryptococcal diseases were excluded from analysis. This study was approved by the ethics committee of the second hospital of Nanjing (reference number 2019-LS-ky013).

### CrAg Lateral Flow Assay

CrAg lateral flow assay was performed per manufacturer's instructions. Patients were firstly screened with qualitative procedure in which undiluted serum samples were mixed with same volume of specimen diluent. When qualitative procedure gave positive result, sample was further measured with semi-quantitative titration procedure that started with an initial dilution of 1:5, followed by 1:2 serial dilutions to 1:2560. In the case of positive qualitative test result but negative semi-quantitative test result, the serum CrAg titer was defined as 1:2. If the specimen is positive at 1:2560, the serum CrAg titer was recorded as ≥1:2560 in clinical lab, however, it was defined as 1:2560 during statistical analysis in this study.

### Case Definitions

Cryptococcal diseases were defined as clinical conditions in which *Cryptococcus* spp. related lesions (such as CM, PC, cryptococcal lymphadenopathy or other cryptococcal lesions) or cryptococcemia were identified. Severe cryptococcal diseases were referred to as CM or cryptococcemia. For simplicity, PC without co-existing CM or cryptococcemia was defined as localized PC. Isolated (or asymptomatic) cryptococcal antigenemia was used to describe the condition that cryptococcal diseases were not found in patients with cryptococcal antigenemia.

A “proven” case of CM should meet one or more of the following criteria: positive cerebrospinal fluid (CSF) India ink staining, positive CSF cryptococcal culture, or positive CSF CrAg test. Cases of PC were classified into three categories, namely “proven,” “probable,” and “possible” PC. “Proven” cases of PC were defined as those in which cryptococcal elements were identified by staining or culture of pleural fluid or lung tissue. “Probable” cases of PC were those with characteristic pulmonary radiological features and cryptococcal elements were identified from sputum or broncholavage fluid. PC was also considered “probable” if patients with cryptococcal antigenemia had characteristic pulmonary radiological features which responded to anti-cryptococcal therapy.

If treatment responses were uncertain in patients with characteristic pulmonary radiographic features and cryptococcal antigenemia, PC was considered “possible.” Possible cases of PC were also discussed by expert panel to evaluate whether those lesions could be explained by infection with other pathogens. Here, the characteristic radiographic features were pulmonary nodules/masses with or without cavitation, as we and others found that those lesions were the most common radiological features of PC in immunocompromised HIV-infected patients (Hu et al., 2013, 2017; Xie et al., 2015). If other forms of PC lesions existed, they were generally accompanied by co-existing pulmonary nodules/masses in our practice (Hu et al., 2017). Therefore, calculating those characteristic lesions may underestimate the prevalence of PC, however, in a mild degree.

### Statistical Analysis

Variables were compared across groups using the Mann–Whitney *U*-test for continuous variables and Chi-Square test for categorical variables. For exploring risk factors associated with positive serum CrAg test, variables of interest included age, sex, antiretroviral therapy (ART) status and CD4 cell count. In addition, serum CrAg titer was also included in analyzing risk factors associated with CM. Adjusted analysis was conducted using binary logistic regression. In multivariable analysis, we included variables that had *priori P-*value < 0.2 in univariable analysis. Statistical analysis was done by SPSS version 22.0 (IBM). A *P-*value <0.05 is considered statistically significant.

## Results

### Prevalence and Risk Factors of Cryptococcal Antigenemia

A total of 872 hospitalized HIV-infected patients were screened for serum CrAg, 21% of whom were on ART with median (interquartile range, IQR) ART duration of 6 (Pac et al., 2015; Rajasingham et al., 2017) months. The prevalence of cryptococcal antigenemia in the study population was 10.3% (90/872; 95% CI, 8.3–12.3%). In unadjusted analysis, there was no difference in risk of cryptococcal antigenemia according to age, sex and ART status (Table 1). Risk of cryptococcal antigenemia was significantly increased for patients with CD4 cell counts < 100 cells/uL (OR 2.98; 95% CI, 1.35, 6.57; *P* = 0.005). The prevalence of cryptococcal antigenemia in patients with CD4 cell counts < 100 cells/ul and between 100 and 199 cell/ul were 11.7% (95% CI, 9.3–14.1%) and 4.3% (95% CI, 1.1–7.4%), respectively. After adjusted for ART status, the CD4 cell counts < 100 cells/uL had 2.84-fold (95% CI, 1.27–6.30; *p* = 0.011) risk to have positive serum CrAg tests.

**Table 1:** Bivariate comparison of risk factors associated with positive serum cryptococcal antigen tests in hospitalized HIV-infected patients.

**Characteristic**	**Overall** **(*n* = 872)**	**Positive sCrAg** **(*n* = 90)**	**Negative sCrAg** **(*n* = 782)**	**OR (95% CI)**	***P*-value**
Age, median years (IQR)	41 (30, 52)	40 (29,49)	41 (30,52)	NA	0.328
Male sex (%)	804(92.2)	84 (93.3)	720 (92.1)	1.21 (0.51–2.87)	0.672
Not on ART (%)	689 (79.0)	76 (84.4)	613 (78.4)	1.50 (0.83–2.71)	0.182
CD4 cell count <100 cells/uL	708 (81.2)	83 (92.2)	625 (79.9)	2.98 (1.35–6.57)	0.005

*sCrAg, serum cryptococcal antigen; OR, odds ratio; CI, confidence interval; IQR, interquartile range; NA, not applicable*.

### Cryptococcal Antigenemia Related Clinical Cryptococcal Diseases

Of the 90 patients with cryptococcal antigenemia (qualitative procedure), 89 patients had serum CrAg titer results and were included for analysis of clinical cryptococcal diseases. Of these 89 patients, 87.6% (95% CI, 80.7–94.6%) had clinical cryptococcal diseases; 12.4% (95% CI, 5.4–19.3%) had isolated cryptococcal antigenemia. The prevalence of CM, cryptococcemia and PC were 58.4% (95% CI, 48.0–68.9%), 50.7% (95% CI, 39.1–62.2%) and 68.5% (95% CI, 58.7–78.4%), respectively (Table 2). One patient (1.1%) had pathologically confirmed cryptococcal lymphadenopathy. Severe cryptococcal diseases (including 3 non-meningeal cryptococcemia with co-existing PC and 52 cases of CM) was identified in 61.8% (95% CI, 51.5–72.1%) of the patients with cryptococcal antigenemia, while localized PC was identified in 25.8% (95% CI, 16.6–35.1%).

**Table 2:** Cryptococcal diseases in hospitalized HIV-infected patients with cryptococcal antigenemia.

**Cryptococcal antigenemia (*n* = 89)^*a*^**				
	**Cryptococcal meningitis**	**Cryptococcemia**	**Pulmonary cryotococcosis**	**Isolated antigenemia**
Total evaluated case	89	75	89	89
Diagnosed case	52	38	61	11
Proven	52	38	7	NA
Probable	NA	NA	45^*b*^	NA
Possible	NA	NA	9	NA
Prevalence	58.4%	50.6%	68.5%	12.4%

*NA, not applicable*.

a *Serum cryptococcal antigen titer was unavailable in one of the 90 patients screened positive for cryptococcal antigenemia (using qualitative procedure). This patient was excluded from analysis of the cryptococcal diseases*.

b *Cryptococcal elements were identified from respiratory samples in three patients. Of those three patients, one had positive staining and culture of the Cryptococcus spp. from bronchoalveolar lavage fluid (BALF), one had cryptococcal antigen and nuclear acids detected from BALF, and the other one had positive culture of the Cryptococcus spp. from sputum. Cryptococcal antigen from the BALF was detected with lateral flow assay, and nuclear acids identification was done using metagenomic next-generation sequencing*.

The distributions of age, sex, ART status and CD4 cell count were similar between CM and non-meningeal PC (P>0.05), and the proportion of patients with missing data regarding cryptococcemia were comparable between the two groups (5/52 and 5/26, respectively; *P* = 0.594). When the patients with missing data were excluded from analysis, cryptococcemia occurred in 74.5% (35/47) of the patients with CM and 14.3%(3/21) of the patients with non-meningeal PC, respectively (χ^2^ = 21.3, *p* < 0.001).

### Distribution of Serum CrAg Titers in Different Cryptococcal Diseases

Of the 89 patients with serum CrAg titer results, the median (range) serum CrAg titer was 1:320 (1:2–1:2560). The median (range) serum CrAg titers in patients with severe cryptococcal diseases (*n* = 55), localized PC (*n* = 23) and isolated cryptococcal antigenemia (*n* = 11) were 1:2560 (1:10–1:2560), 1:20 (1:2–1:320) and 1:5 (1:2–1:320), respectively. Serum CrAg titers in severe cryptococcal diseases were significantly higher than those in localized PC or isolated antigenemia (Mann–Whitney U = 108, n1 =55, n2 = 34, *P* < 0.001). There were no statistically significant differences in serum CrAg titers between localized PC and isolated antigenemia (Mann–Whitney U = 95, n1 =23, n2 = 11, *P* =0.240). 75 % (39/52) of the patients with CM and 78.9% (30/38) of the patents with cryptococcemia had serum CrAg titers ≥1:640. 82.6% (19/23) of the patients with localized PC and 90.9% (10/11) of the patients with isolated cryptococcal antigenemia had serum CrAg titers ≤ 1:40.

In unadjusted analysis, there was no difference in risk of CM among patients with cryptococcal antigenemia according to sex and ART status (*P* = 0.200 and 0.915, respectively, Table 3). The proportions of patients with an age of ≤ 45 years or CD4 cell count of <50 cells/mL were higher in CM patients; however, the differences did not achieve statistical significance (*P* = 0.087 and *P* = 0.051, respectively). Serum CrAg titers ≥1:320 were strongly associated with CM (OR 26.88; 95%CI, 8.36–86.42; P < 0.001). Multivariable analysis demonstrated that serum CrAg titer was the only risk factor for CM. After adjusted for age and CD4 cell count, patients with serum CrAg titers ≥1:320 had 25.88-fold (95% CI, 7.65–6.30; *p* = 0.011) risk to have CM.

**Table 3:** Bivariate comparison of risk factors associated with cryptococcal meningitis in hospitalized HIV-infected patients with cryptococcal antigenemia.

**Characteristic**	**Overall** **(*n* = 89)**	**Cryptococcal meningitis (*n* = 52)**	**Non-cryptococcal meningitis (*n* = 37)**	**OR (95% CI)**	***P*-value**
Age ≤ 45y (%)	55 (61.8)	36 (69.2)	19 (51.4)	2.13 (0.89–5.10)	0.087
Male sex (%)	83 (93.3)	47 (90.4)	36 (97.3)	0.26 (0.03–2.33)	0.200
Not on ART (%)	75 (84.3)	44 (84.6)	31 (83.8)	0.94 (0.30–2.98)	0.915
CD4 cell count <50 cells/uL(%)	65 (73.0)	42 (80.8)	23 (62.2)	2.56 (0.98–6.66)	0.051
sCrAg titers ≥1:320(%)	47 (52.8)	42 (80.8)	5 (13.5)	26.88 (8.36–86.42)	<0.001
sCrAg titers ≥1:640(%)	40 (44.9)	39 (75.0)	1 (2.7)	108 (13.4–867.8)	<0.001

*sCrAg, serum cryptococcal antigen; OR, odds ratio; CI, confidence interval; IQR, interquartile range*.

When serum CrAg titers were divided into low (≤ 1:20), median (1:40 1:160) and high (≥1:320) titers, the proportion of patients had CM were 12% (3/25), 41.2% (7/17), and 89.4% (42/47), respectively (χ2 =42.784; *p* < 0.001). All the patients with serum CrAg titers ≥1:640 had severe cryptococcal diseases (39 CM and 1 non-meningeal cryptococcemia). CM occurred in 25.9% (7/27) of the patients with serum CrAg titers between 1:10 and 1:80. None of the patients with serum CrAg titers ≤ 1:5 had CM, although 45.5% (5/11) of whom had PC.

## Discussion

A recent meta-analysis showed that the pooled prevalence of cryptococcal antigenemia in hospitalized HIV-infected patients was 9.8% (Ford et al., 2018). Our study in Chinese population had comparable finding that the prevalence was 10.3% (Ford et al., 2018). Of the parameters analyzed, lower CD4 cell count was the only risk factor associated with cryptococcal antigenemia in our study. After adjusted for ART status, CD4 cell counts < 100 cells/uL had 2.84-fold risk to have cryptococcal antigenemia compared with those between 100 and 199 cells/uL. Nevertheless, even in patients with CD4 cell counts between 100 and 199 cells/uL, the prevalence of cryptococcal antigenemia was relatively high (4.3%) exceeding threshold necessitating routine CrAg screening (Rajasingham et al., 2012, 2019). Those results supported routine CrAg screening in hospitalized HIV-infected patients with CD4 cell counts <200 cells/uL.

The vast majority of the patients (87.6%) with cryptococcal antigenemia in our study had clinically relevant cryptococcal diseases. Underlying pulmonary involvement occurred in 68.5% of the patients with cryptococcal antigenemia, which was much higher than previously thought (32% of the inpatients) (Meyohas et al., 1995). An important reason for this discrepancy may be that chest CT scan, which was more sensitive to reveal pulmonary lesions compared with chest radiograph, was routinely performed for patients with cryptococcal antigenemia in our study. As we and others had previously described, the most characteristic radiological features of HIV-associated PC were pulmonary nodules/masses with or without cavitation that may not always be associated with significant respiratory symptoms; clinicians may be unawareness of PC when pulmonary symptoms were not severe (Hu et al., 2013, 2017; Xie et al., 2015). Together, our study suggested that the prevalence of PC in HIV-infected patients may be underestimated.

Previous reported central nervous system (CNS) involvement ranged from 71.3 to 88.9% in hospitalized HIV-infected patients with cryptococcal antigenemia (Wajanga et al., 2011; Mamuye et al., 2016; Chen et al., 2019), and from 19 to 67% in outpatients (Beyene et al., 2017; Wake et al., 2019). In a recent meta-analysis, the pooled prevalence of CM among CrAg-positive outpatients was 33% (95% CI, 21–45%) (Temfack et al., 2019), which was much lower than that in our study. This suggested that inpatients may have more profound cryptococcal diseases compared with outpatients. Nevertheless, in our study, routine cryptococcal antigenemia screening still identified about 40% of the cryptococcal infection before invading the CNS. Of note, PC contributed substantially to non-meningeal cryptococcal antigenemia. For HIV-infected patients with cryptococcal antigenemia, we recommend perform both lumbar puncture and chest CT scan to evaluate possible CNS and respiratory system cryptococcal diseases.

Recent Ethiopian CrAg screening program showed that nearly all HIV-infected patients (97%; 28/29) with plasma CrAg titers ≥1:640 had CM. None of the patients with CrAg titers ≤ 1:80 had CM (Beyene et al., 2017). We found similar result that all the patients with serum CrAg titers ≥1:640 had severe cryptococcal diseases (including 39 CM and 1 non-meningeal cryptococcemia). However, about a quarter of the patients with serum CrAg titers between 1:10 and 1:80 had CM. We recommended that HIV-infected patients with CD4 cell counts <200 cell/ul and serum CrAg titers ≥1:640 should be treated as life-threatening cryptococcal diseases when they are reluctant to lumbar puncture and/or blood culture was unavailable. When the serum CrAg titers ≤ 1:5, lumbar puncture or blood culture may be optional as the risk of CM was very low. However, chest CT scan still needed to be performed as almost half of those patients may have PC.

In our study, 82.6% (19/23) of the patients with localized PC had serum CrAg titers ≤ 1:40. The relatively lower serum CrAg titers in those patients may reflect early pulmonary cryptococcal infection with low fungal burden. Lack of high serum CrAg titers in those immunocompromised patients perhaps implied that cryptococci may early disseminate and invade the CNS system before they substantially multiply and cause extensive damage in the lung. Similar with localized PC, isolated cryptococcal antigenemia was also associated with lower serum CrAg titers. Isolated cryptococcal antigenemia may reflect early or latent cryptococcal infection in which current diagnostic strategies were insensitive to detect any underlying lesions. In this condition, pre-emptive treatment should be administered to prevent life-threatening CM, just as current guidelines recommended (WHO, 2018). However, isolated cryptococcal antigenemia may be also related to a recovery stage of past cryptococcal infection therefore antifungal treatment may not be needed. This hypothesis was supported by the findings in a prospective study on CrAg screening that some of the HIV-infected patients with untreated asymptomatic antigenemia (possibly with low serum CrAg titers) remained alive and none developed CM at 1 year (Longley et al., 2016).

In conclusion, we found a cryptococcal antigenemia prevalence of 10.3% in hospitalized HIV-infected patients. The majority of the hospitalized HIV-infected patients with cryptococcal antigenemia already developed clinical cryptococcal diseases most commonly involving the lung. Routine screening for cryptococcal antigenemia identified about 40% of the cryptococcal infection before progressing to CM. Serum CrAg titers are correlated with the severity of untreated cryptococcal diseases. This may potentially serve as a marker to guide initial anti-fungal treatment.

## Data Availability Statement

The datasets generated for this study are available on request to the corresponding author.

## Ethics Statement

The studies involving human participants were reviewed and approved by The ethics committee of the second hospital of Nanjing. The patients/participants provided their written informed consent to participate in this study.

## Author Contributions

ZH designed this study. MX, ZH, and WC collected the data. MX, ZP, CX, ZH, and WC analyzed the data. YChe, YChi, HW, and ZH were involved in the clinical management of the patients and analyzed the clinical data. This manuscript was initially drafted by MX, ZH, and WC and then revised by other authors in this study. All authors approved the final manuscript.

## Conflict of Interest

The authors declare that the research was conducted in the absence of any commercial or financial relationships that could be construed as a potential conflict of interest.

## Abbreviations

HIV: human immunodeficiency virus
CM: cryptococcal meningitis
CrAg: cryptococcal antigen
PC: pulmonary cryptococcosis
CSF: cerebrospinal fluid
ART: antiretroviral therapy
CNS: central nervous system
IQR: interquartile range
CI: confidence interval.
